# The moderating effect of emotion regulation in the association between coping strategies and resilience in Lebanese adults

**DOI:** 10.1186/s40359-022-01019-9

**Published:** 2022-12-09

**Authors:** Emmanuelle Awad, Mirna Fawaz, Souheil Hallit, Sahar Obeid

**Affiliations:** 1 Social and Education Sciences Department, School of Arts and Sciences, Lebanese American University, Jbeil, Lebanon; 2 Faculty of Health Sciences, Beirut Arab University, Tareek Al Jadida, Afeef Al Tiba, 1105 Beirut, Lebanon; 3 School of Medicine and Medical Sciences, Holy Spirit University of Kaslik, P.O. Box 446, Jounieh, Lebanon; 4 Research Department, Psychiatric Hospital of the Cross, Jal Eddib, Lebanon; 5 Applied Science Research Center, Applied Science Private University, Amman, Jordan

**Keywords:** Resilience, Coping strategies, Emotion regulation, Lebanon

## Abstract

**Objectives:**

To evaluate the psychometric properties of the Coping Strategies Inventory–Short Form and investigate the relationship between coping strategies and resilience, taking into consideration the moderating role of emotion regulation.

**Methods:**

This cross-sectional study was carried out between May and July 2022. A total of 387 participants was recruited through convenience sampling through several areas in Lebanon governorates. The data was collected through an online questionnaire containing the following sections: sociodemographic information about the participants, Connor-Davidson Resilience Scale (CD-RISC), Emotion Regulation Questionnaire and Coping Strategies Inventory–Short Form.

**Results:**

The confirmatory factor analysis results confirmed the four-factor structure of the Coping Strategies Inventory–Short Form, with no measurement invariance across gender. In individuals with high problem-focused engagement and emotion-focused engagement, lower expressive suppression was significantly associated with more resilience. In individuals with high problem-focused disengagement, having high expressive suppression was significantly associated with less resilience.

**Conclusion:**

The current study provides novel and distinct findings regarding the relationship between emotion regulation, coping strategies and resilience in all of their dimensions. Furthermore, the present results provide insight on how a population under extreme stress receives and reacts to its reality.

## Background

Emotion regulation isn’t just the ability to control or change one’s emotions but the capacity to handle experiences that may be stressful or arousing in a manner that is effective with a flexible range of adaptive emotions [1]. Two strategies related to emotion regulation have been identified: reappraisal, which is the modification of an experience’s meaning in order to change the emotional response eventually and suppression, which consists of inhibiting behavioral and emotional reactions to a certain experience [2]. These two strategies have different timings as reappraisal happens at the beginning of emotion generation while suppression happens after the emotions are generated [3].

Emotion regulation is also the capacity to guide emotions in both negative experiences that trigger stress and anxiety as well as positive experiences that produce joy and excitement [4]. In contrast, coping strategies are actions that are employed to manage and reduce negative emotions that arise from negative experiences [5]. Similarly to emotion regulation, coping strategies also have two dimensions that can be differentiated: engagement coping strategies meant to restrict the effects of a negative experience in order to protect psychological wellbeing while disengagement coping strategies aim to avert a negative experience and decrease its negative effects [6]. It is important to note that engagement coping strategies are more effective in promoting psychological wellbeing but disengagement coping strategies are associated with worse psychological wellbeing according to the literature [6]. In a previous study, a notable observation was made: the type of coping strategy employed varied according to the problem encountered [7]; this introduces the problem-focused coping strategy, which is investigated in this study. Problem-focused coping consists of making efforts and behaving in a way that stop or change negative situations [8]. On the other hand, emotion-focused coping targets efforts towards stopping or changing emotional responses to negative events [8].

Within the same realm, resilience can be defined as a measure to deal with negative experiences such as stress and anxiety, and therefore can be considered as a tactic to cope [9]. In essence, resilience is the capacity to proceed adaptively despite encountering a negative experience [10]. Previous research has shown that resilience is relative, with some people having better abilities to deal with adversity and subsequently increasing the probability of surviving effectively and thriving in life [11]. In this context, it has been concluded previously that coping strategies significantly promote higher resilience [12]. Inversely, implicating resilience-focused programs increased the employment of effective coping strategies [13]. Furthermore, emotion regulation in a way that cultivates positive emotions increased resilience in the future when encountering negative situations [14]. It has been suggested previously that the association between emotion regulation and resilience has been overlooked [15], suggesting that the relationship needs to be further explored. More specifically, reappraisal was associated with resilience as individuals who scored high on reappraisal were more likely to be resilient to psychological stress [16]. Furthermore, positive cognitive reappraisal was positively correlated with resilience [17]. As for the suppression dimension, it was negatively correlated with resilience [18].

Some evidence suggests that emotion regulation and coping strategies overlap in some aspects while still remaining independent [19]. Another study found that problems in emotion regulation were a predictor of all dimensions of coping strategies including problem-focused and emotion-focused engagement and disengagement [20], indicating that failure to engage in emotion regulation might have been associated with employment of coping strategies. Findings suggest that achieving progress on both emotion regulation and coping strategies is essential for avoiding or reducing the negative effects of stressful situations [21], further confirming the benefits of emotion regulation for psychological wellbeing. Within the same context, emotion regulation moderated the relationship between stress and wellbeing [22]. Emotion regulation also played a moderating role between maladaptive psychological variables, anxiety and insomnia [23]. These results suggest that emotion regulation could potentially be a moderator between various psychological variables.

Emotion regulation, coping strategies and resilience are tactics used to deal with experiences of all kinds. When evaluating the situation of the Lebanese people, it is more than obvious that the experience faced by the collective is extremely negative. On top of that, this small country has been recently paralyzed by many tragedies including the COVID-19, and the fourth of August Beirut port explosion, the world’s most powerful non-nuclear explosion [24]. In a study done on 988 Lebanese citizens, findings showed that 63% of Lebanese young adults are highly dissatisfied with their country, which cripples their flourishing in life [25], as opposed to individuals living in European or Northern American regions where life is possibly less challenging. Furthermore, Lebanon has been going through the worse economic crisis in its modern history with the rapid deterioration of the national currency, one of the highest inflation rates in the world and the lack of resources such as electricity and fuel. Not only does it make the topic of this study interesting but could provide insight on how the Lebanese people are still able to move forward with their lives in an adaptive way that maintains relative psychological wellbeing. For that reason, the aim of the current study was to evaluate the psychometric properties of the Coping Strategies Inventory – Short Form and investigate the relationship between coping strategies and resilience, taking into consideration the moderating role of emotion regulation.

## Methods

### Study design

This cross-sectional study was carried out between May and July 2022, enrolling a total of 387 persons. The research team initiated the contact with friends and family members they know; those people were asked to forward the link to their friends and family members. The link was shared among participants and sent to all districts/governorates of Lebanon (Beirut, Mount Lebanon, North Lebanon, South Lebanon, and Bekaa) through social networks, using the snowball technique. Excluded were those who refused to fill out the questionnaire. There were no fees for participating in the study.

### Minimal sample size calculation

According to the G-power software [26], a minimum of 316 students was deemed necessary to have enough statistical power, based on a 5% risk of error, 80% power, f^2^ = 2.5% and 10 factors to be entered in the multivariable analysis.

### Questionnaire

The questionnaire used was anonymous and in Arabic, the native language in Lebanon; it required approximately 10 to 15 min to complete. The questionnaire consisted of three parts. The first part of the questionnaire included an explanation of the study topic and objective, a statement ensuring the anonymity of respondents. The participant had to select the option stating *I consent to participate in this study* to be directed to the questionnaire.

The second part of the questionnaire contained sociodemographic information about the participants (age, gender, region of living, marital status and education level). The Household Crowding Index (HCI), reflecting the socioeconomic status of the family [27], is the ratio of the number of persons living in the house over the number of rooms in it (excluding the kitchen and the bathrooms).

The third part included the scales used in this study:

#### Connor-Davidson resilience scale (CD-RISC)

The CD-RISC, validated in Lebanon [28], comprises 10 items [9, 10], each of which are scored on a 5-point scale ranging from 0 (*not true at all*) to 4 (*true nearly all of the time*). Examples of items include, “I am able to adapt when changes occur” and “I am not easily discouraged by failure.” Higher scores on the CD-RISC-10 indicate higher levels of resilience. In this study, the Cronbach’s alpha value was 0.88.

#### Emotion regulation questionnaire

Validated in Lebanon [29], it is composed of 10 items that measure whether a respondent uses cognitive reappraisal or expressive suppression to regulate their emotions. Answers options varied between 1 (strongly disagree) and 7 (strongly agree). Higher scores reflect a larger use of the concerned emotion regulation strategy [30]. In this study, the Cronbach’s alpha values were 0.91 for the cognitive reappraisal subscale and 0.84 for the expressive suppression subscale.

#### Coping strategies inventory–short form (CSI-SF)

The 16-item CSI-SF evaluates four coping strategies defined by two binary dimensions: problem-focused vs. emotion-focused and engagement vs. disengagement [31]. These strategies are combined into four subscales: problem-focused engagement; problem-focused disengagement; emotion-focused engagement; and emotion-focused disengagement. Answers options varied between 1 = “Never”, 2 = “Seldom”, 3 = “Sometimes”, 4 = “Often” and 5 = “Almost Always”. Higher scores reflect a larger use of the concerned coping strategy. In this study, the Cronbach’s alpha values were as follows: problem-focused engagement (0.83), problem-focused disengagement (0.78), emotion-focused engagement (0.73) and emotion-focused disengagement (0.75).

### Translation procedure

The forward and backward translation method was applied to the CSI-SF. The English version was translated to Arabic by a Lebanese translator who was completely unrelated to the study. Afterwards, a Lebanese psychologist with a full working proficiency in English, translated the Arabic version back to English. The initial English version and the second English version were compared to detect and later eliminate any inconsistencies.

### Statistical analysis

We had no missing data since all questions were required. A confirmatory factor analysis (CFA) was administered based on maximum likelihood estimation in SPSS AMOS v.24. For this purpose, the normed model chi-square (χ²/df), the Steiger-Lind root mean square error of approximation (RMSEA), the Tucker-Lewis Index (TLI) and the comparative fit index (CFI). Values ≤ 3 for χ²/df, and ≤ 0.06 for RMSEA, and 0.90 for CFI and TLI indicate good fit of the model to the data [32, 33].

To examine gender invariance of coping strategies scores, we conducted multi-group CFA [34] using the total sample. Measurement invariance was assessed at the configural, metric, and scalar levels [35]. Configural invariance implies that the latent coping strategies variable(s) and the pattern of loadings of the latent variable(s) on indicators are similar across gender (i.e., the unconstrained latent model should fit the data well in both groups). Metric invariance implies that the magnitude of the loadings is similar across gender; this is tested by comparing two nested models consisting of a baseline model and an invariance model. Lastly, scalar invariance implies that both the item loadings and item intercepts are similar across gender and is examined using the same nested-model comparison strategy as with metric invariance [34]. Following the recommendations of Cheung and Rensvold [36] and Chen [34], we accepted ΔCFI ≤ 0.010 and ΔRMSEA ≤ 0.015 or ΔSRMR ≤ 0.010 (0.030 for factorial invariance) as evidence of invariance [37].

The SPSS software v.25 was used for the statistical analysis. Cronbach’s alpha values were calculated for all scales and subscales. The resilience score was considered normally distributed since the skewness (= 0.096) and kurtosis (=-0.252) values varied between − 1 and + 1 [38]. The Student t was used to compare two means and the Pearson test was used to correlate two continuous variables. The moderation analysis was conducted using PROCESS MACRO v3.4, model 1 taking each coping strategy score as an independent variable, cognitive reappraisal/expressive suppression as moderators and resilience as the dependent variable. Results adjusted over age, gender, marital status, education level and household crowding index. *p* < .05 was deemed statistically significant.

## Results

### Sociodemographic and other characteristics of the sample

Three hundred eighty-seven participants participated in this study, with a mean age of 26.17 ± 11.47 years and 58.4% females. Other descriptive statistics of the sample can be found in Table 1.

**Table 1:** Sociodemographic and other characteristics of the sample (N = 387)

Variable	N (%)
Sex	
Male	161 (41.6%)
Female	226 (58.4%)
Marital status	
Single	311 (80.4%)
Married	76 (19.6%)
Education level	
Secondary or less	66 (17.1%)
University	321 (82.9%)
Region of living	
Urban	294 (76.0%)
Rural	93 (24.0%)

	Mean ± SD
Age (years)	26.17 ± 11.47
Household crowding index (persons/room)	1.47 ± 1.00
Resilience	23.88 ± 7.29
Problem-focused engagement	12.70 ± 3.46
Problem-focused disengagement	10.60 ± 3.28
Emotion-focused engagement	12.47 ± 3.29
Emotion-focused disengagement	11.47 ± 3.25
Cognitive reappraisal	23.83 ± 8.36
Expressive suppression	16.56 ± 5.51

### Confirmatory factor analysis (CFA) of the coping strategies scale

The CFA indicated that fit of the four-factor model of the coping strategies scale was acceptable: χ^2^/df = 332.07/98 = 3.39, RMSEA = 0.079 (90% CI 0.069, 0.088), SRMR = 0.063, CFI = 0.902, TLI = 0.880. The standardized factor loading are summarized in Table 2.

**Table 2:** Items of the short form of the Coping Strategies Inventory in English and Standardized Estimates of Factor Loadings from the Confirmatory Factor Analysis (CFA) in the total sample

Item	CFA
Problem-focused engagement	
5	0.72
6	0.76
11	0.73
13	0.74
Problem-focused disengagement	
1	0.69
2	0.75
8	0.67
9	0.67
Emotion-focused engagement	
4	0.57
7	0.66
12	0.64
14	0.68
Emotion-focused disengagement	
3	0.47
10	0.79
15	0.77
16	0.62

### Gender invariance

As reported in Table 3, all indices suggested that configural, metric, and scalar invariance was supported across gender.

**Table 3:** Measurement invariance across gender in the total sample

Model	χ^2^	*df*	CFI	RMSEA	SRMR	Model comparison	Δχ^2^	ΔCFI	ΔRMSEA	ΔSRMR	Δ*df*	*p*
Configural	553.17	196	0.866	0.067	0.079							
Metric	549.75	208	0.864	0.065	0.080	Configural vs. metric	3.42	0.002	0.002	0.001	12	0.991
Scalar	564.12	220	0.864	0.064	0.079	Metric vs. scalar	14.37	< 0.001	0.001	0.001	12	0.277

*CFI* comparative fit index, *RMSEA* Steiger-Lind root mean square error of approximation, *SRMR* Standardised root mean square residual

### Bivariate analysis of factors associated with resilience

The results of the bivariate analysis of factors associated with resilience are summarized in Tables 4 and 5. The results showed that higher problem- and emotion-focused engagement and cognitive reappraisal were significantly associated with more resilience, whereas higher problem- and emotion-focused disengagement, and expressive suppression were significantly associated with less resilience.

**Table 4:** Bivariate analysis of factors associated with resilience and post-traumatic growth

Variable	Resilience score (mean ± SD)	*p*	Post-traumatic growth (mean ± SD)	*p*
Sex		0.131		0.707
Male	24.54 ± 7.69		27.78 ± 11.59	
Female	23.40 ± 6.98		28.23 ± 11.93	
Marital status		0.108		0.296
Single	24.17 ± 7.34		28.35 ± 11.70	
Married	22.67 ± 7.04		26.78 ± 12.09	
Education level		0.130		0.114
Secondary or less	22.64 ± 7.09		25.95 ± 11.99	
University	24.13 ± 7.32		28.47 ± 11.71	
Region of living		0.301		0.959
Urban	23.66 ± 6.98		28.06 ± 11.34	
Rural	24.56 ± 8.20		27.99 ± 13.14

**Table 5:** Correlations of continuous variables with resilience

	1	2	3	4	5	6	7	8	9
1. Resilience	1								
2. Problem-focused engagement	0.26***	1							
3. Problem-focused disengagement	− 0.43***	− 0.57***	1						
4. Emotion-focused engagement	0.20***	0.62***	− 0.49***	1					
5. Emotion-focused disengagement	− 0.25***	− 0.36***	0.47***	− 0.59***	1				
6. Cognitive reappraisal	0.47***	0.33***	− 0.47***	0.23***	− 0.22***	1			
7. Expressive suppression	− 0.41***	− 0.17**	0.38***	− 0.23***	0.37***	− 0.74***	1		
8. Age	− 0.07	0.02	− 0.02	− 0.02	0.04	− 0.03	0.01	1	
9. Household crowding index	− 0.09	0.03	− 0.07	− 0.003	0.04	− 0.08	0.09	0.13*	1

*p < 0.05; **p < 0.01; ***p < 0.001

### Moderation analysis with resilience taken as the dependent variable

**Fig. 1:**
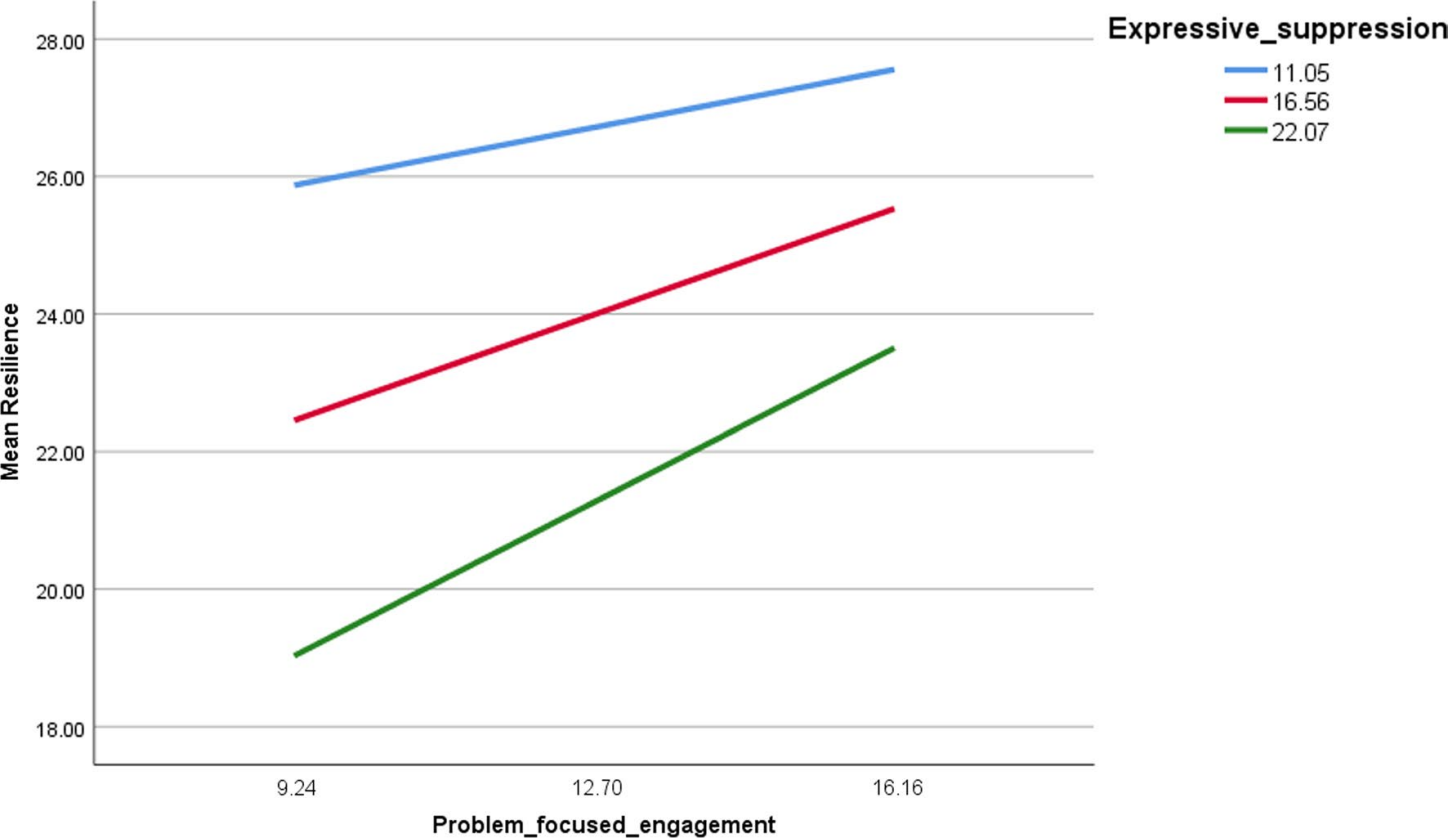
Moderation of expressive suppressive in the association between problem-focused engagement and resilience

**Fig. 2:**
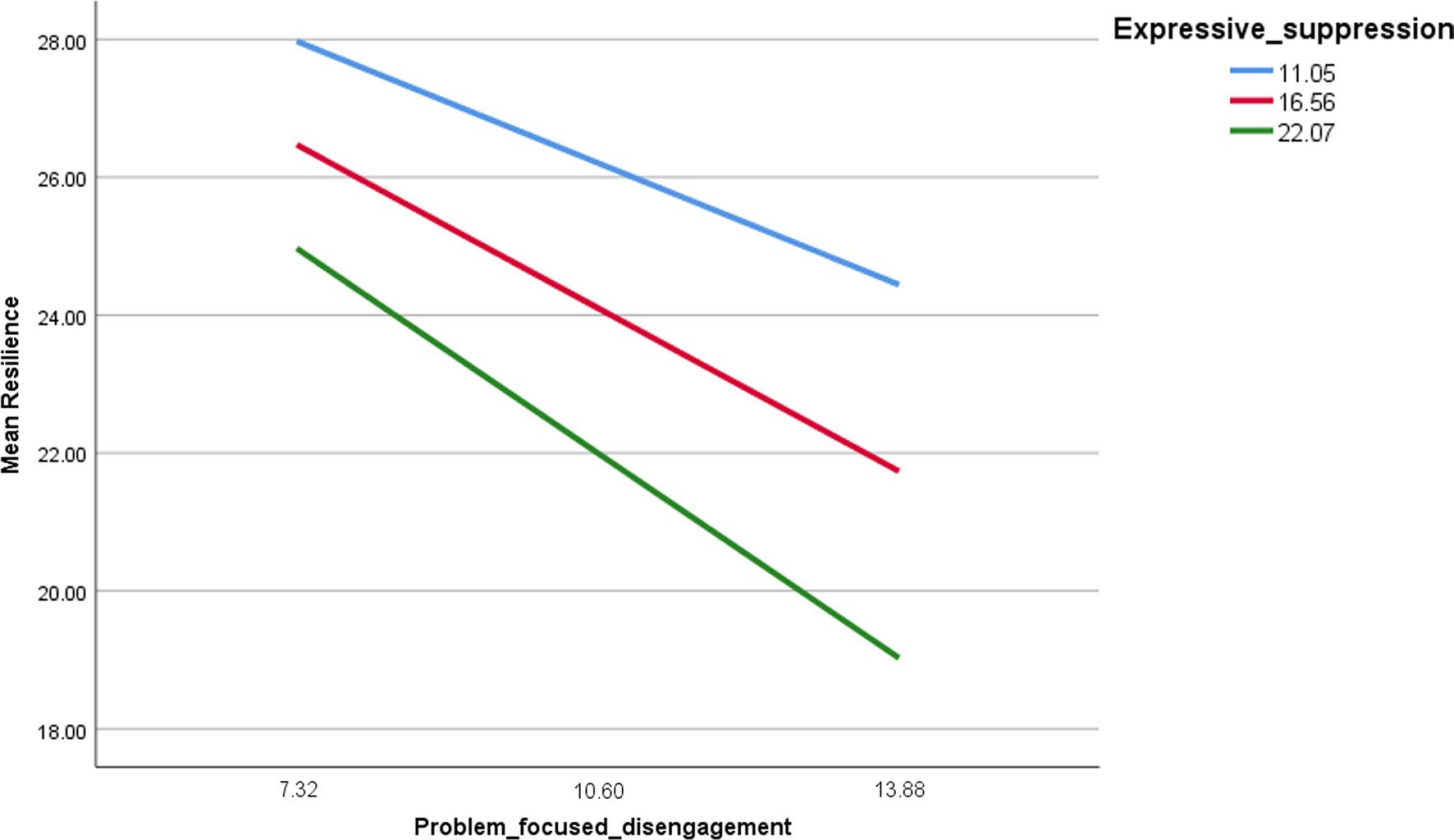
Moderation of expressive suppressive in the association between problem-focused disengagement and resilience

**Fig. 3:**
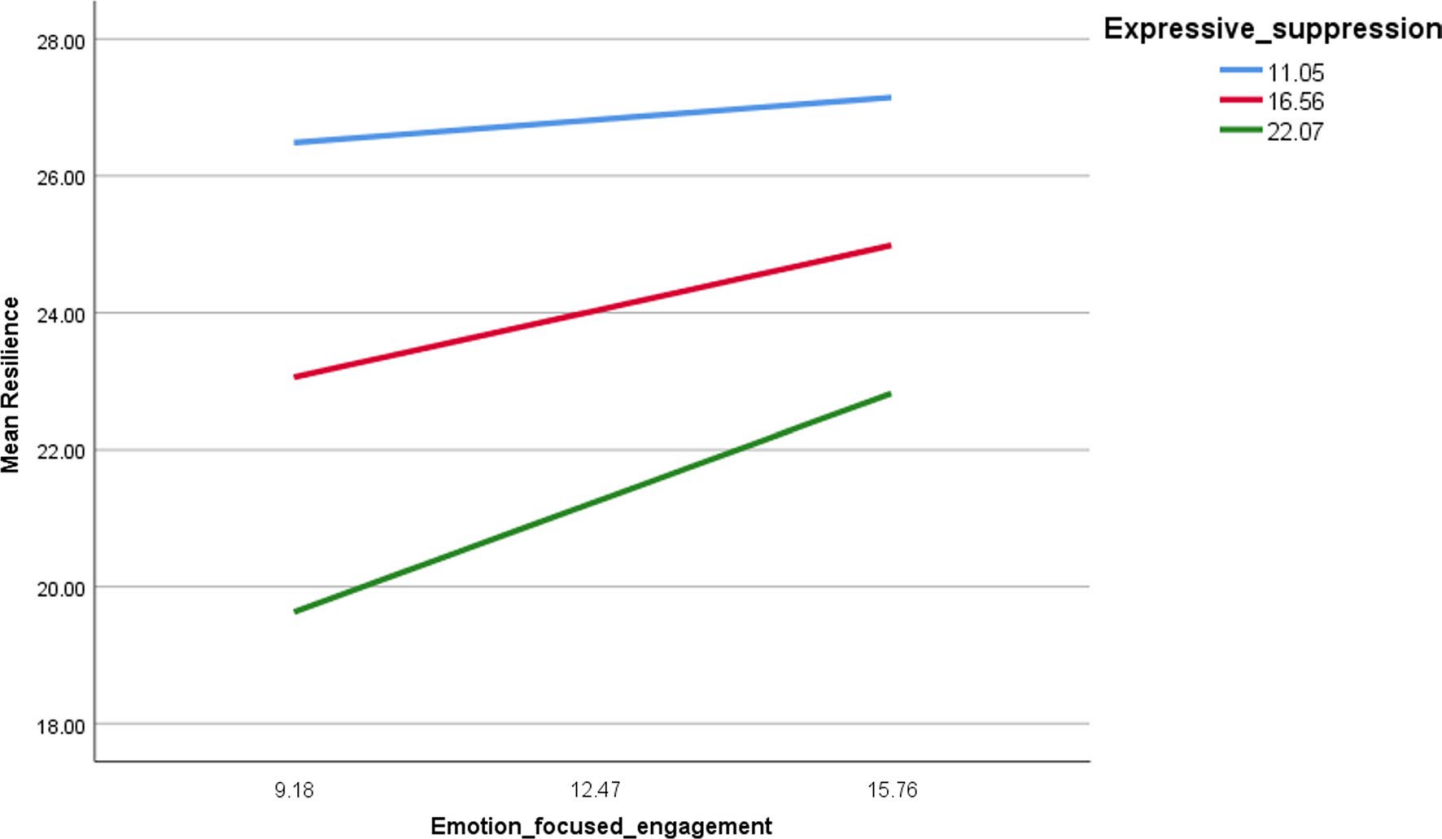
Moderation of expressive suppressive in the association between emotion-focused engagement and resilience

The details of the moderation analysis of cognitive reappraisal/expressive suppression taken as moderators in the associations between coping strategies and resilience, are summarized in Table 6. In persons with high problem-focused engagement (Fig. 1), lower expressive suppression was significantly associated with more resilience at low (Beta = 0.24, t = 2.007, *p* = 0.045), moderate (Beta = 0.45, t = 4.540, *p* < 0.001) and high (Beta = 0.65, t = 4.589, *p* < 0.001) expressive suppression levels. Moreover, in persons with high problem-focused disengagement, having high expressive suppression was significantly associated with less resilience (Fig. 2) at low (Beta = − 0.54, t = 3.788; *p* < .0.01), moderate (Beta = − 0.72, t = − 6.80,  *p* < .0.001) and high (Beta = − 0.91; t = − 6.477, *p* < 0.001) levels of expressive suppression levels. In persons with high emotion-focused engagement (Fig. 3), lower expressive suppression was significantly associated with more resilience at moderate (Beta = 0.29, t = 2.715, *p* = 0.007) and high (Beta = 0.49, t = 3.025, *p* = 0.003) levels of expressive suppression levels.

**Table 6:** Moderation analysis taking each coping strategy score as an independent variable, cognitive reappraisal/expressive suppression as moderators and resilience as the dependent variable

	Beta	t	*p*	95% CI
Model 1: cognitive reappraisal as the moderator				
Problem-focused engagement	− 0.02	-1.39	0.167	− 0.04; 0.01
Problem-focused disengagement	0.01	0.50	0.618	− 0.02; 0.03
Emotion-focused engagement	− 0.02	− 1.61	0.109	− 0.04; 0.004
Emotion-focused disengagement	0.01	0.57	0.568	− 0.02; 0.03
Model 2: expressive suppression as the moderator				
Problem-focused engagement	0.04	2.30	0.022	0.01; 0.07*
Problem-focused disengagement	− 0.03	− 1.98	0.049	− 0.07; − 0.001*
Emotion-focused engagement	0.04	1.99	0.048	0.001; 0.07*
Emotion-focused disengagement	− 0.003	− 0.17	0.867	− 0.04; 0.03

*Indicates significant moderation; results adjusted over age, gender, marital status, education level and household crowding index

## Discussion

### Role of emotion regulation in general

It has been previously established that cultural particularity plays a monumental role in the emotional regulation strategies that are adopted by individuals [39]. A previous study assessing emotion regulation between two different countries found that the Lebanese sample’s emotion regulation tendencies reflected collectivistic values and approaches emphasizing social interaction [39]. In comparison, the other sample from the United Kingdom showed less emotion focus, such as emotion-focused engagement, which was perceived as typical for societies where individualism is prevalent and social relationships are less common than in Lebanon [39]. Overall, differences in emotion regulation are present even among countries from the same region such as Northern, Eastern and Southern Europe [40]. Such emotion regulation variations can even be found between Western Asian and Eastern Asian countries [41]. This further confirms that emotion regulation varies cross-culturally.

### Moderating effect of emotion regulation between problem- and emotion-focused engagement and resilience

In individuals with high problem-focused engagement and emotion-focused engagement, lower expressive suppression was significantly associated with more resilience. Problem-focused engagement refers to taking action to resolve a problem [42] and minimize the psychological repercussions of a negative experience. Meanwhile, emotion-focused engagement consists of efforts to alter emotional responses towards certain events with the aim of minimizing the negative effects caused by it. A previous study found that focus on both problem-focused regulation and emotion-focused regulation produces optimal results when it comes to emotion regulation interventions [43]. Research has shown that active strategies such as engagement emotion regulation are more likely to produce positive psychological outcomes and less likely to produce negative psychological outcomes [42]. Furthermore, higher suppression was connected with less responsive behavior [44], which is in accordance with the current results where lower suppression is connected to higher engagement. Therefore, it can be inferred that proactive behavior such as problem-focused engagement, emotion-focused engagement and less suppression are more likely to be associated with more resilience, which have a higher probability of protecting psychological wellbeing.

### Moderating effect of emotion regulation between problem-focused disengagement and resilience

Moreover, in individuals with high problem-focused disengagement, having high expressive suppression was significantly associated with less resilience. Problem-focused disengagement can be defined as aiming to solve a problem through the avoidance of a negative situation. Disengagement was found to be less effective in problem solving and subsequently less likely to produce positive outcomes [45]. Also, higher expressive suppression was associated with negative emotional consequences [46]. Similarly, another study showed that suppression was related to higher negative emotion and lower positive emotion [30]. More importantly for the current study, suppression was associated with ineffective regulation of emotions according to previous results [47]. As can be deduced from the results discussed, disengagement and suppression have a positive relationship and both are related to worse psychological outcomes. This can be considered in accordance with the current results where high problem-focused engagement and high suppression were related to less resilience. It was found that engaging in positive emotions is associated with developing higher resilience when encountering negative situations [14]. Furthermore, effective emotion regulation was positively correlated with resilience [48]. As previously mentioned, disengagement strategy for emotion regulation are less effective and associated with worse psychological variables [6]. Resilience is an adaptive tactic to deal with negative experiences and therefore, it can be considered rational that our study results indicated that problem-focused disengagement and high expressive suppression, which are related to worse psychological outcomes, are associated with less resilience.

### Clinical implications

Emotion regulation can significantly influence how individuals react to daily life events, whether positive or negative. The current results shed light on the nature of the relationship between emotion regulation, coping strategies and resilience, which in turn help clinicians determine how a certain population most likely reacts to experiences. Subsequently, practitioners are able to tailor interventions that would be most effective for the Lebanese population, in this case. Furthermore, they might be able to identify how certain profiles react to negative situations based on the variables at hand and avoid unpleasant responses such as violent or emotionally charged ones.

### Limitations

First, the data was collected through an online survey, which might produce response bias. Second, the current study is cross-sectional and therefore causation cannot be inferred regarding the relationship between variables. Third, the lack of studies assessing the moderating role of emotion regulation between coping strategies and resilience affects the ability to compare the current results with previous ones. In addition, information bias might occur due to the use of self-report measures to assess the variables evaluated. Furthermore, despite the fact that some fit indices in the CFA results might have a poor fit to the data, these cut-off values should not be interpreted rigidly [49, 50] and values between 3.01 and 5.00 for χ_normed_^2^ and between 0.08 and 0.10 for RMSEA can indicate acceptable but mediocre fit to the data [51, 52]. Accordingly, the Arabic version of the short form of the coping strategies inventory might be considered a valid instrument for the assessment of coping strategies among Lebanese adults. Finally, it is important to mention the possibility of residual confounding bias because other variables that might affect resilience were not considered in this study.

## Conclusion

The current study provides findings regarding the relationship between emotion regulation, coping strategies and resilience in all of their dimensions. Not only does this introduce novel results but also offers precise inferences relating to each emotion regulation aspects, different coping strategies and distinct elements of resilience within the Lebanese population. Furthermore, the present results provide insight on how a population under extreme stress receives and reacts to its reality.

## Future perspectives

This study is the first of its kind within the Lebanese population, to the best of our knowledge. For that reason, it might inspire the replication of such an investigation involving different populations, especially in countries in the region that don’t suffer from the same stressors as the Lebanese ones. This is important in order to inquire more about the role of the environment as well as the individual characteristics of people. Additionally, a longitudinal study on the same population could clarify the variability of such strategies and reactions as situations change and progress.

## Data Availability

All data generated or analyzed during this study are not publicly available due the restrictions from the ethics committee. However, all the datasets are available from corresponding author on reasonable request.
